# Impact of acquisition time and penalizing factor in a block-sequential regularized expectation maximization reconstruction algorithm on a Si-photomultiplier-based PET-CT system for ^18^F-FDG

**DOI:** 10.1186/s13550-019-0535-4

**Published:** 2019-07-24

**Authors:** Elin Trägårdh, David Minarik, Helén Almquist, Ulrika Bitzén, Sabine Garpered, Erland Hvittfelt, Berit Olsson, Jenny Oddstig

**Affiliations:** 1 Clinical Physiology and Nuclear Medicine, Skåne University Hospital, Inga Marie Nilssons gata 49, 205 02 Malmö, Sweden; 2 Wallenberg Center for Molecular Medicine, Lund University, Lund, Sweden; 3 Radiation Physics, Skåne University Hospital, Malmö and Lund, Sweden; 4 Nuclear Medicine, Lund University, Malmö, Sweden

**Keywords:** PET-CT, FDG, Image reconstruction, Q. Clear, Block-sequential regularized expectation maximization

## Abstract

**Background:**

Block-sequential regularized expectation maximization (BSREM), commercially Q. Clear (GE Healthcare, Milwaukee, WI, USA), is a reconstruction algorithm that allows for a fully convergent iterative reconstruction leading to higher image contrast compared to conventional reconstruction algorithms, while also limiting noise. The noise penalization factor *β* controls the trade-off between noise level and resolution and can be adjusted by the user. The aim was to evaluate the influence of different *β* values for different activity time products (ATs = administered activity × acquisition time) in whole-body ^18^F-fluorodeoxyglucose (FDG) positron emission tomography with computed tomography (PET-CT) regarding quantitative data, interpretation, and quality assessment of the images.

Twenty-five patients with known or suspected malignancies, referred for clinical ^18^F-FDG PET-CT examinations acquired on a silicon photomultiplier PET-CT scanner, were included. The data were reconstructed using BSREM with *β* values of 100–700 and ATs of 4–16 MBq/kg × min/bed (acquisition times of 1, 1.5, 2, 3, and 4 min/bed). Noise level, lesion SUV_max_, and lesion SUV_peak_ were calculated. Image quality and lesion detectability were assessed by four nuclear medicine physicians for acquisition times of 1.0 and 1.5 min/bed position.

**Results:**

The noise level decreased with increasing *β* values and ATs. Lesion SUV_max_ varied considerably between different *β* values and ATs, whereas SUV_peak_ was more stable. For an AT of 6 (in our case 1.5 min/bed), the best image quality was obtained with a *β* of 600 and the best lesion detectability with a *β* of 500. AT of 4 generated poor-quality images and false positive uptakes due to noise.

**Conclusions:**

For oncologic whole-body ^18^F-FDG examinations on a SiPM-based PET-CT, we propose using an AT of 6 (i.e., 4 MBq/kg and 1.5 min/bed) reconstructed with BSREM using a *β* value of 500–600 in order to ensure image quality and lesion detection rate as well as a high patient throughput. We do not recommend using AT < 6 since the risk of false positive uptakes due to noise increases.

## Background

^18^F-fluorodeoxyglucose (FDG) positron emission tomography with computed tomography (PET-CT) is a powerful and widely used imaging technique primarily employed in oncology. Recently, a novel generation of PET scanners based on silicon (Si)-photomultiplier (PM) technology was introduced that can potentially increase pathology detection, primarily due to its higher sensitivity [1–3]. At the same time, improved reconstruction methods have been developed, for example, the block-sequential regularized expectation maximization algorithm (BSREM) [4] with the commercial name Q. Clear (GE Healthcare, Milwaukee, WI, USA) [2, 5]. This method allows for fully convergent iterative reconstruction, resulting in higher image contrast compared to ordered subset expectation maximization (OSEM) while also reducing noise. With conventional reconstruction algorithms, the accuracy of the measured standardized uptake value (SUV) in lesions improves when the number of iterations is increased but this also increases the noise level. Stopping the iterative process after a limited number of iterations, to reduce noise, can lead to an underestimation of SUV in smaller lesions. The BSREM algorithm suppresses noise via a penalty factor *β*, where higher values suppress noise more, but also reduce the resolution. Using the BSREM algorithm increases the convergence of SUV, particularly in small lesions, and improves quantitative accuracy compared to OSEM in phantom studies and with simulated lesions [4, 6–10]. Lesion detectability with BSREM has also been found to be equal to or higher compared to OSEM [10, 11].

The impact of the *β* factor has been investigated by others for ^18^F-fluciclovine [12], ^68^Ga-prostate-specific membrane antigen [13], and ^18^F-FDG [4, 14–16]. However, previous studies on ^18^F-FDG have only investigated image quality and lesion detectability for acquisition times of 3–4 min/bed position [4, 14] with an activity administration of 4 MBq/kg, which may not be a clinically relevant acquisition time for nuclear medicine departments aiming at an increased throughput.

The aim of the present study was to evaluate the influence of different activity time products (ATs = administered activity × acquisition time) and different *β* values (range 100–700) in whole-body ^18^F-FDG SiPM-based PET-CT regarding quantitative data as well as visual interpretation and quality assessment.

## Materials and methods

### PET-CT system

Three Discovery MI (GE Healthcare, Milwaukee, WI, USA) PET-CT systems were used for image acquisition. The systems were configured with four rings of detector blocks with lutetium yttrium oxyorthosilicate crystals (crystal size 4.0 × 5.3 × 25 mm^3^) coupled to an array of SiPM. The PET detector has a transaxial field of view of 70 cm, an axial field of view of 20 cm, and an overlap of 24% between multi-bed positions (per vendor recommendation). The sensitivity, according to NEMA standards, was 13 cps/kBq. The PET-CTs were cross-calibrated to the dose calibrator, and the calibration is validated monthly using an SUV control with phantoms. The PET system was combined with a 128-slice CT.

### Patients and imaging

This study included 25 patients aged 18 years or more referred for clinical ^18^F-FDG PET-CT due to known or suspected malignancies at Skåne University Hospital between 30 April and 20 June 2018 (lung cancer *n* = 11, colorectal cancer *n* = 3, esophageal cancer *n* = 2, cholangiocarcinoma *n* = 2, breast cancer *n* = 1, malignant melanoma *n* = 1, testicular cancer *n* = 1, sarcoma *n* = 1, vulvar cancer *n* = 1, tonsil cancer *n* = 1, and giant cell carcinoma *n* = 1). In total, 40% (*n* = 10) of the patients were women. The mean (± SD) weight was 70 ± 11 kg (range 44–92 kg), the mean BMI was 23.6 ± 3.5 (range 16.5–29.7), the mean age was 59 ± 14 years (range 24–81 years), and the mean glucose level was 102.6 ± 21.6 mg/dL (range 75.6–172.8).

Imaging was performed 60 min after administration, and the patients were scanned from the inguinal region to the base of the skull. The mean administrated ^18^F-FDG was 4.0 ± 0.1 MBq/kg (range 3.8–4.3 MBq/kg), and the mean accumulation time was 63 ± 4 min (range 55–74 min). The PET images were reconstructed using the commercially available BSREM algorithm Q. Clear (GE Healthcare, Milwaukee, WI, USA) including the time-of-flight and point spread functions with a 256 × 256 matrix (pixel size 2.7 × 2.7 mm^2^, slice thickness 2.8 mm). The patients were examined with an acquisition time of 4 min/bed in list mode. Sinograms with acquisition times of 1, 1.5, 2, 3, and 4 min/bed were created from the list files, and these were reconstructed with seven different *β* values, 100, 200, 300, 400, 500, 600, and 700, thus yielding 35 image series per patient.

Due to unexpected uptakes visible on 1 min/bed images but not on 4 min/bed images (see the “Results” section) in a couple of the patients, the images from these patients were also reconstructed with a OSEM algorithm (4 iterations, 16 subsets, 2 mm post-filter) with time of flight (TOF), one with and one without point spread function (PSF). We also reconstructed the 4-min/bed position acquisition into four different 1-min series (0–1 min, 1–2 min, 2–3 min, and 3–4 min), using BSREM with *β* 500.

The activity and acquisition times are to a close approximation interchangeable, as long as the count rate is within the linear part of the noise-equivalent count rate curve, which is generally the case in clinical 18F-FDG studies [2]: 8 MBq/kg with an acquisition time of 1 min/bed is equivalent to 4 MBq/kg and 2 min/bed, assuming the same accumulation time between administration and scan time (1 h in this study). Therefore, going forward, we will use AT to refer to the product of the administered activity per unit body weight and the acquisition time (MBq/kg × min/bed), to emphasize that the results do not depend on the acquisition time alone but the combination of time and activity.

CT images were acquired for attenuation correction and anatomic correlation of the PET images. A diagnostic CT with intravenous and oral contrast (9 patients) or a low-dose CT without contrast (16 patients) was performed. In our clinical routine, a low-dose CT is performed if a previous diagnostic CT has been performed within 4 weeks. For diagnostic CTs, tube current modulation was applied by adjusting the tube current for each individual with a noise index of 42.25 and a tube voltage of 100 kV. The CT used for attenuation correction was acquired in a late venous phase. For low-dose CT, the tube voltage was 120 kV with a noise index of 45. The adaptive statistical iterative reconstruction technique (ASiR-V) was applied for all CT reconstructions.

This study was approved by the Regional Ethical Review Board (#2016/417) and was performed in accordance with the Declaration of Helsinki. All patients provided written informed consent.

### Image analysis

#### Quantitative analysis

The noise level was calculated from regions of interest (ROIs) in the liver drawn on transaxial images using Hermes 2.0.0 (Hermes Medical Solutions, Stockholm, Sweden). Three 5-cm^2^ ROIs were drawn in subsequent transaxial slices with one image in-between, and these measurements were averaged. None of the ROIs were placed where liver metastases or large vessels were seen. The ROIs were drawn in one image series and copied to the other image series. The noise level was calculated as the ratio between the SUV_standard deviation (SD)_ and the SUV_mean_ in the liver.

Two lesions per patient were selected and marked by an experienced nuclear medicine physician (see details below). SUV_max_ and SUV_peak_ (SUV_mean_ in a 1-cm^3^ volume sphere) were calculated for all lesions.

#### Qualitative analysis

Ten image series were regarded as clinically interesting/relevant with reasonable acquisition times and reasonable image quality (1.5 and 1 min/bed position with *β* values of 300, 400, 500, 600, and 700) and were chosen for further evaluation. Acquisition times of ≥ 2.0 min/bed position were not evaluated since they were regarded as too long for most nuclear medicine departments. *β* values of 100–200 were not evaluated due to noisy images (see the “Results” section below).

Two lesions per patient were selected and marked by an experienced nuclear medicine physician in the image series with 4 min/bed position and a *β* value of 300, which based on visual analysis was regarded as the reference. The lesions chosen were approximately 1 cm in diameter and had slight to moderate hypermetabolism. Preferably, malignant lesions were selected, but if no malignant lesions fitting the criteria were found, reactive or physiological uptakes were chosen. Four nuclear medicine physicians reviewed the reference as well as the ten clinically relevant image series simultaneously and assessed the detectability of the lesions. The detectability was graded on a scale of 1–3, where 1 = lesion is visible, 2 = uncertain if lesion is visible, and 3 = lesion is not visible (compared with local background). The ten image series of clinical relevance were also evaluated for image quality. Four reviewers assessed which image series had acceptable overall image quality based on noise level, artifacts, contrast, and sharpness.

### Statistical analysis

Continuous patient parameters are presented as mean ± SD and range and categorical variables as a percent (%). Noise level, SUV_max_, and SUV_peak_ were tested for normality using the Shapiro-Wilks test. Since the variables were not normally distributed, all quantitative PET data are shown as a median ± interquartile range (IQR). Statistical significance was considered for *p* values less than 0.05, and all statistical tests were performed using IBM SPSS version 25 (IBM, Armonk, NY, USA). Noise level, SUV_max_, and SUV_peak_ were all normalized to the reference (4 min/bed, *β* of 300) in the figures. Lesion detectability was calculated for all of the lesions and all of the observers (two lesions per patient, 25 patients, four observers = 200 assessed lesions in total) and expressed as a percent. The total number of images with acceptable image quality was calculated (one score per patient, 25 patients, four observers = 100 assessments of image quality in total) and expressed as a percent.

## Results

### Image analysis

#### Quantitative analysis

**Fig. 1:**
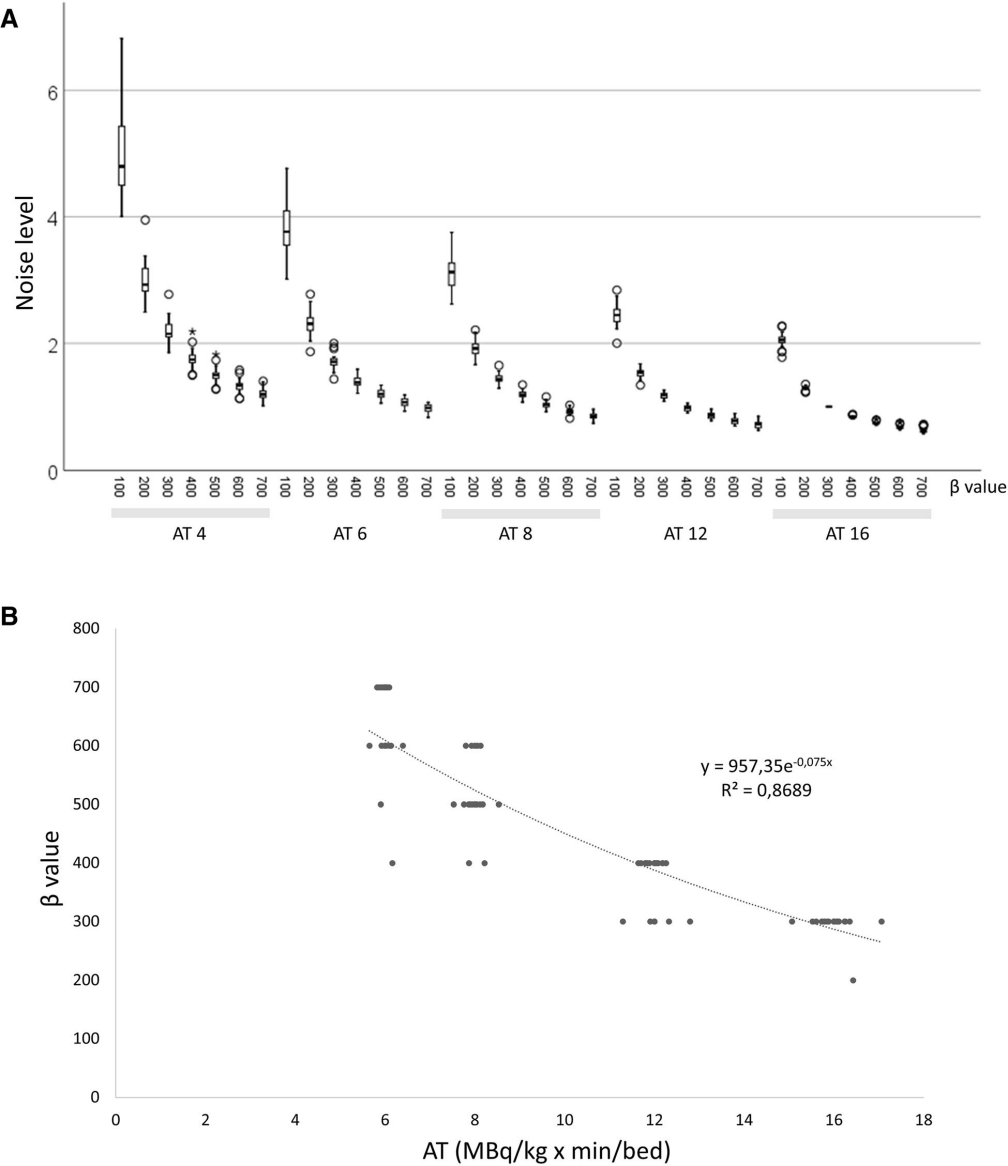
**a** Noise level for all examined combinations of AT (MBq/kg × min/bed) and *β* values. All values are normalized to AT of 16 with a *β* of 300 (reference). The boxplots show the median (thick line in the middle of the boxes) and the first and third quartiles (the top and bottom box lines). The whiskers extend to 1.5 times the height of the box (approx. 95% of the data). Circles are outliers, and stars in the graph indicate extreme outliers. **b** *β* value versus administered activity × acquisition time (AT) (min/bed × MBq/kg). The *β* value for all patients at different AT to obtain the same noise level as for the reference image (AT of 16 with a *β* of 300) (**a**)

The lowest median noise level in the liver was found with an AT of 16 and a *β* of 700 (0.053 ± 0.01, median ± IQR) and the highest with an AT of 4 and a *β* of 100 (0.42 ± 0.09). For each acquisition time, the noise level decreased with increasing *β* values (Fig. 1). The noise level for an AT of 12 with a *β* of 400 (0.084 ± 0.021), AT of 8 with a *β* of 500 (0.089 ± 0.021), and an AT of 6 with a *β* of 700 (0.084 ± 0.018) yielded similar values as the reference series with AT of 16 and *β* of 300 (0.086 ± 0.023). To obtain the same noise level as in the reference image, the *β* could be calculated as a function of activity and time for the special case of 18F-FDG with 60-min accumulation time from Eq. 1 (Fig. 1):

1 $$ \beta =957{e}^{-0.075 AT} $$

where *A* is the administered activity per kilogram of body weight (MBq/kg) and *T* is the acquisition time (min/bed).

**Fig. 2:**
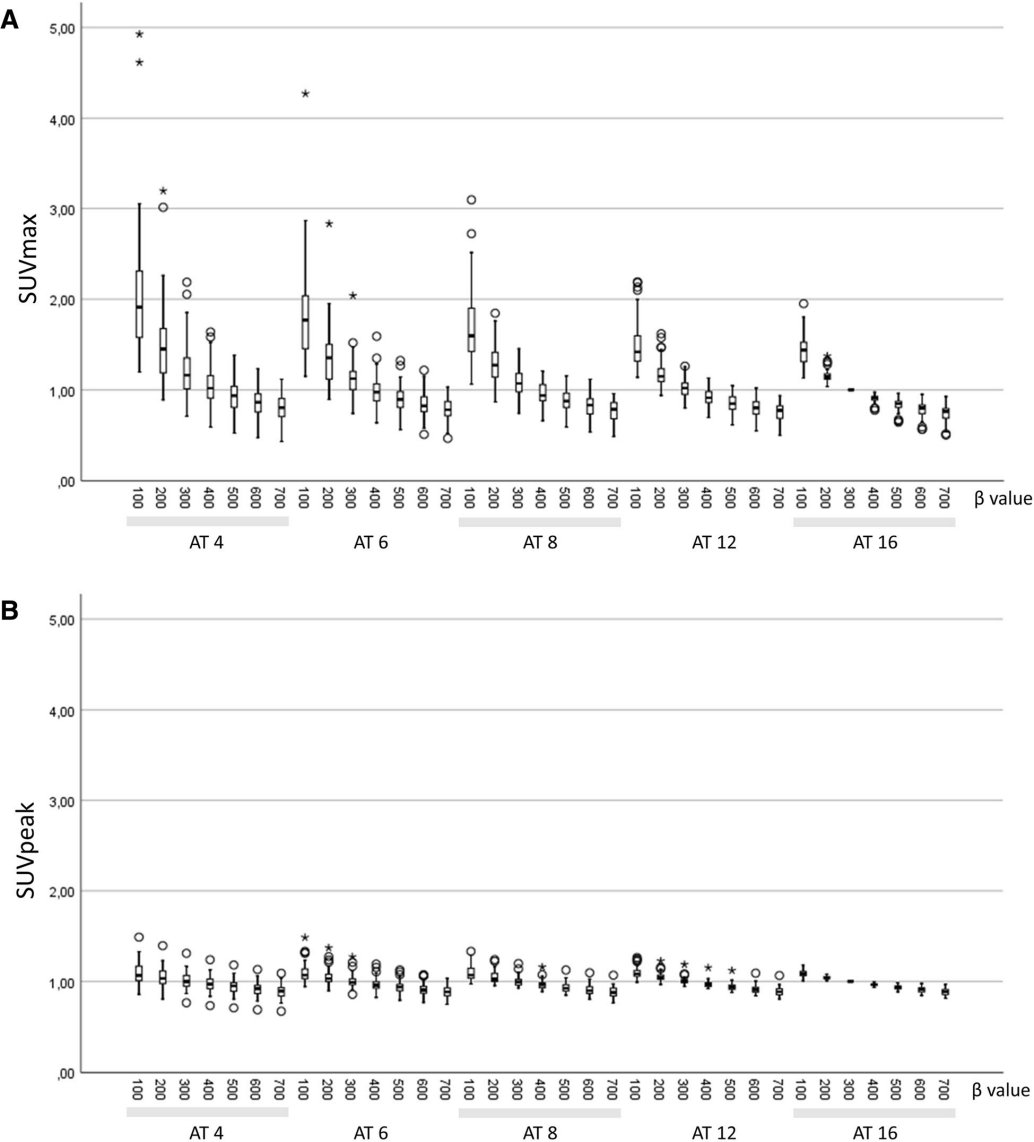
SUV_max_ (**a**) and SUV_peak_ (**b**) for all examined combinations of AT (MBq/kg × min/bed) and *β* values. All values are normalized to an AT of 16 with a *β* of 300 (reference). The same scale on the *y-*axis is used in order to highlight the smaller variation of SUV_peak_ compared with SUV_max_. The boxplots show the median and the second and third quartile groups (box) whereas the whiskers show the first and the fourth quartile groups. Circles are outliers, and stars indicate extreme outliers

The highest median lesion SUV_max_ was found with an AT of 4 and a *β* of 100 (6.7 ± 2.7) and the lowest with an AT of 16 and a *β* of 700 (2.6 ± 1.3). As shown in Fig. 2, SUV_max_ increased with decreasing AT and decreased with increasing *β* values. SUV_peak_ were stable over the range of ATs and decreased less than SUV_max_ with increasing *β* values (Fig. 2).

#### Qualitative analysis

**Fig. 3:**
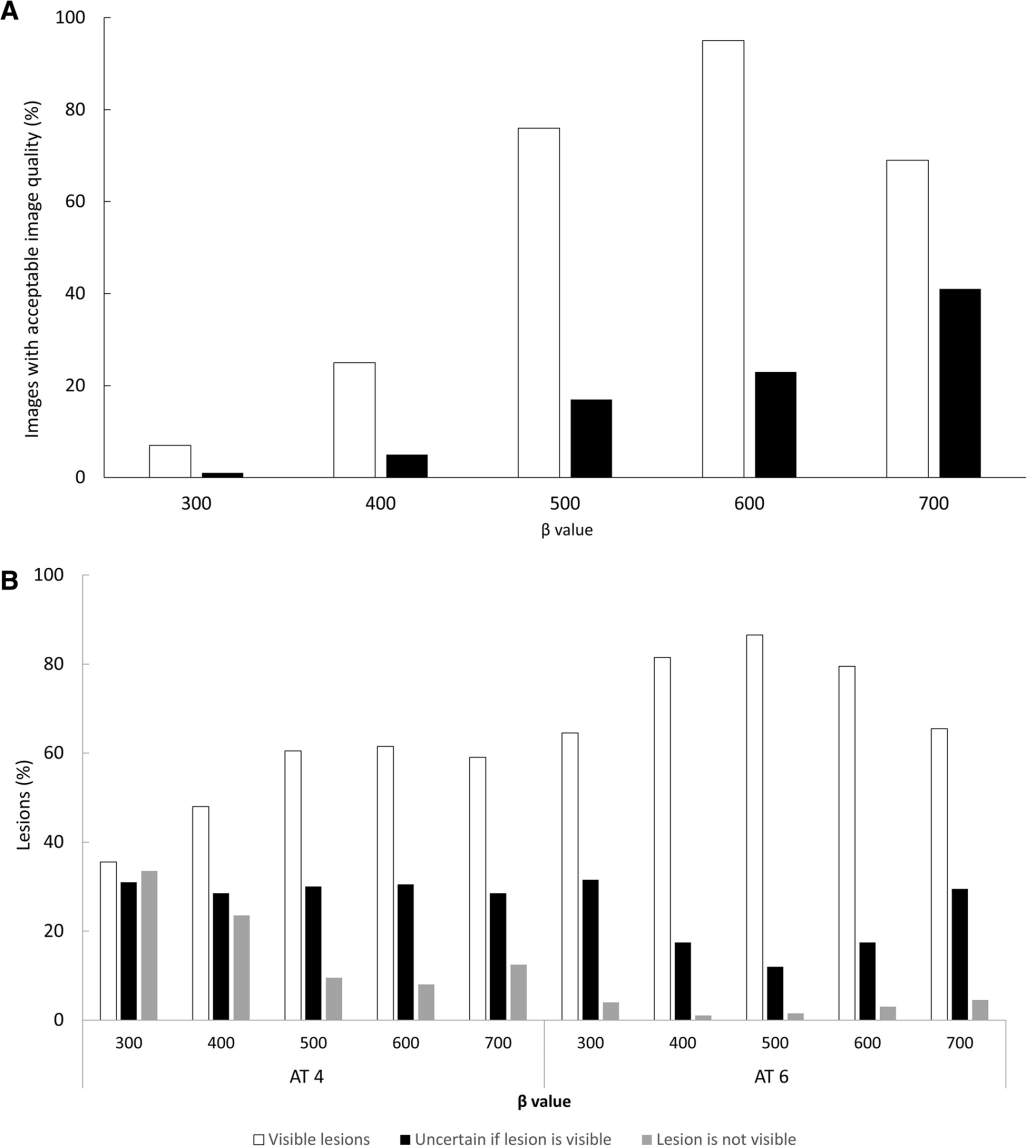
Image quality (**a**) and lesion detectability (**b**) for the combinations of AT (min/bed × MBq/kg) and *β* values

The image series with an AT of 6 with a *β* of 600 yielded the largest percentage of images with acceptable image quality (96% of the images), whereas an AT of 4 with a *β* of 300 had the lowest percentage (1% of the images) (Fig. 3). For an AT of 4, the largest percentage of images with acceptable image quality (41% of the images) was obtained with a *β* of 700.

**Fig. 4:**
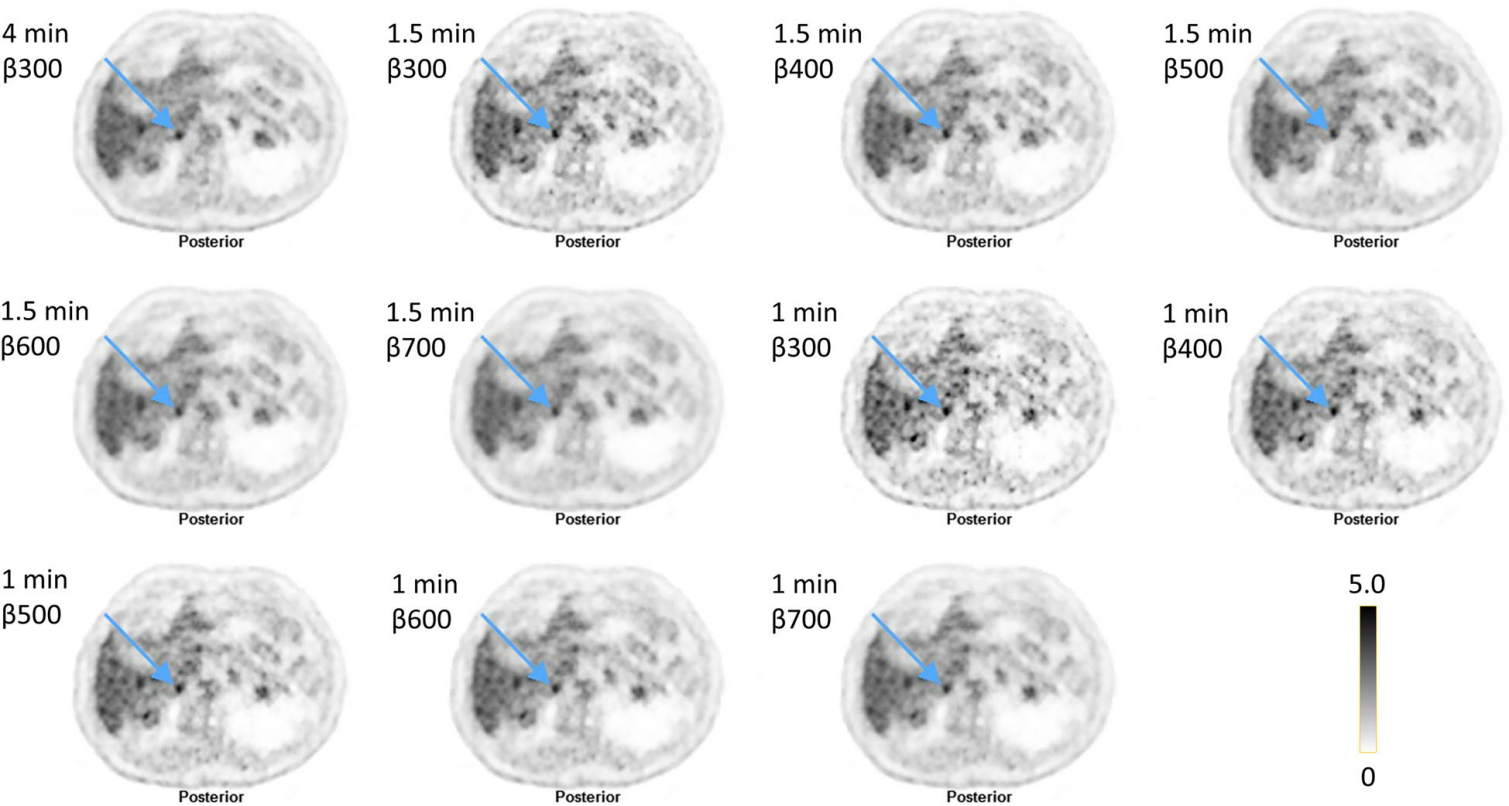
Example of a lesion (right adrenal gland, indicated by the arrow) and how it appears in the reference with an AT of 16 (4 MBq/kg and 4 min/bed position with a *β* of 300) and the combinations of time/bed position and *β* values evaluated

Regarding lesion detectability, an AT of 6 with a *β* of 500 was found to be the best (87% of the lesions were visible, 12% were uncertain, and 2% were not visible) and an AT of 4 with a *β* of 300 was the worst (36% of the lesions were visible, 31% were uncertain, and 34% were not visible) (Fig. 3). Figure 4 shows an example of image quality and lesion detectability.

**Fig. 5:**
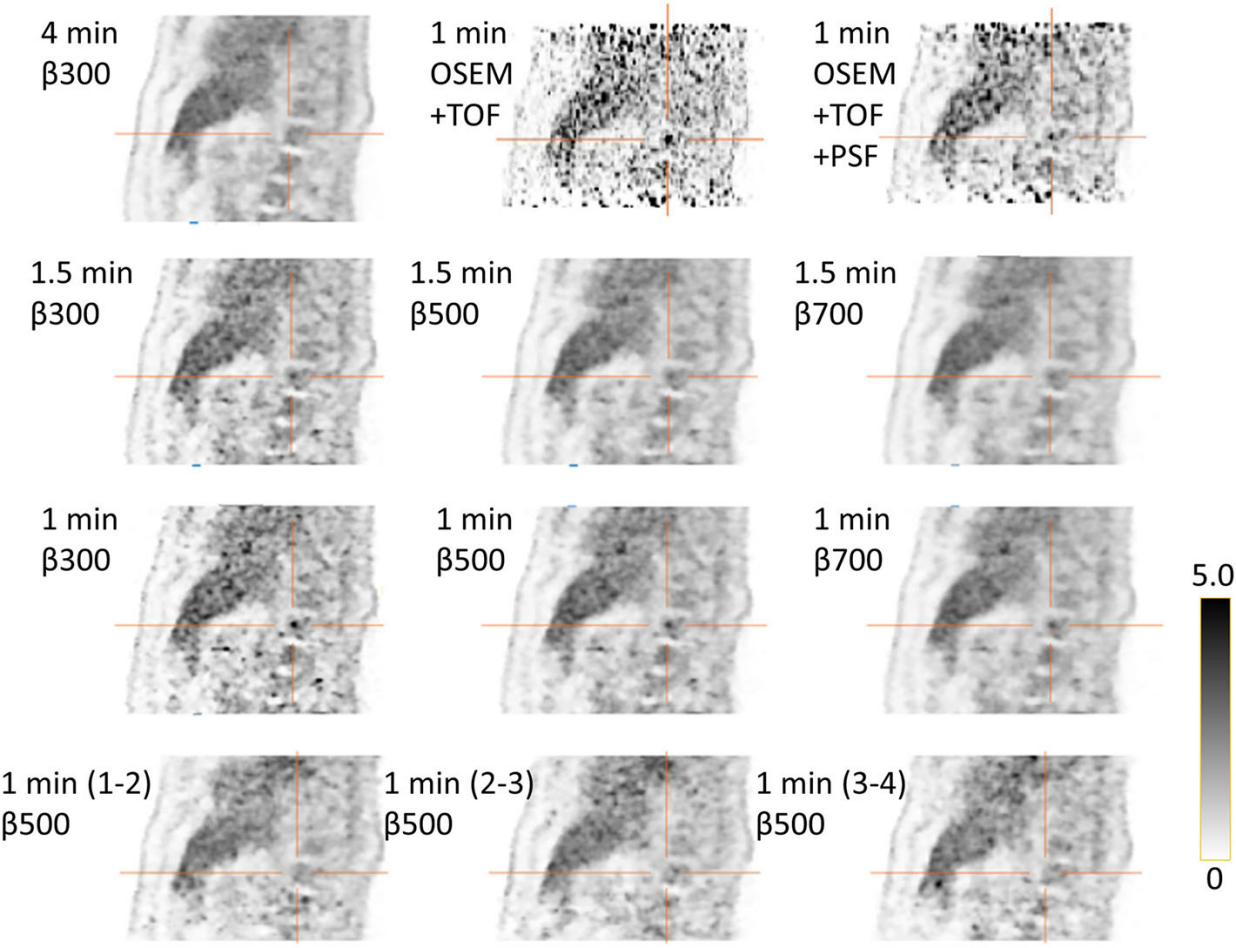
Example of a lesion in a vertebra not visible in the 4-min reference (upper left corner) but, to different degrees, visible in the other combinations of time/bed position and *β* values evaluated. The last row shows 1-min reconstructions from the 4-min acquisition for the last 3 min, where the lesion is not visible. OSEM+TOF with and without PSF are shown in the upper row

**Fig. 6:**
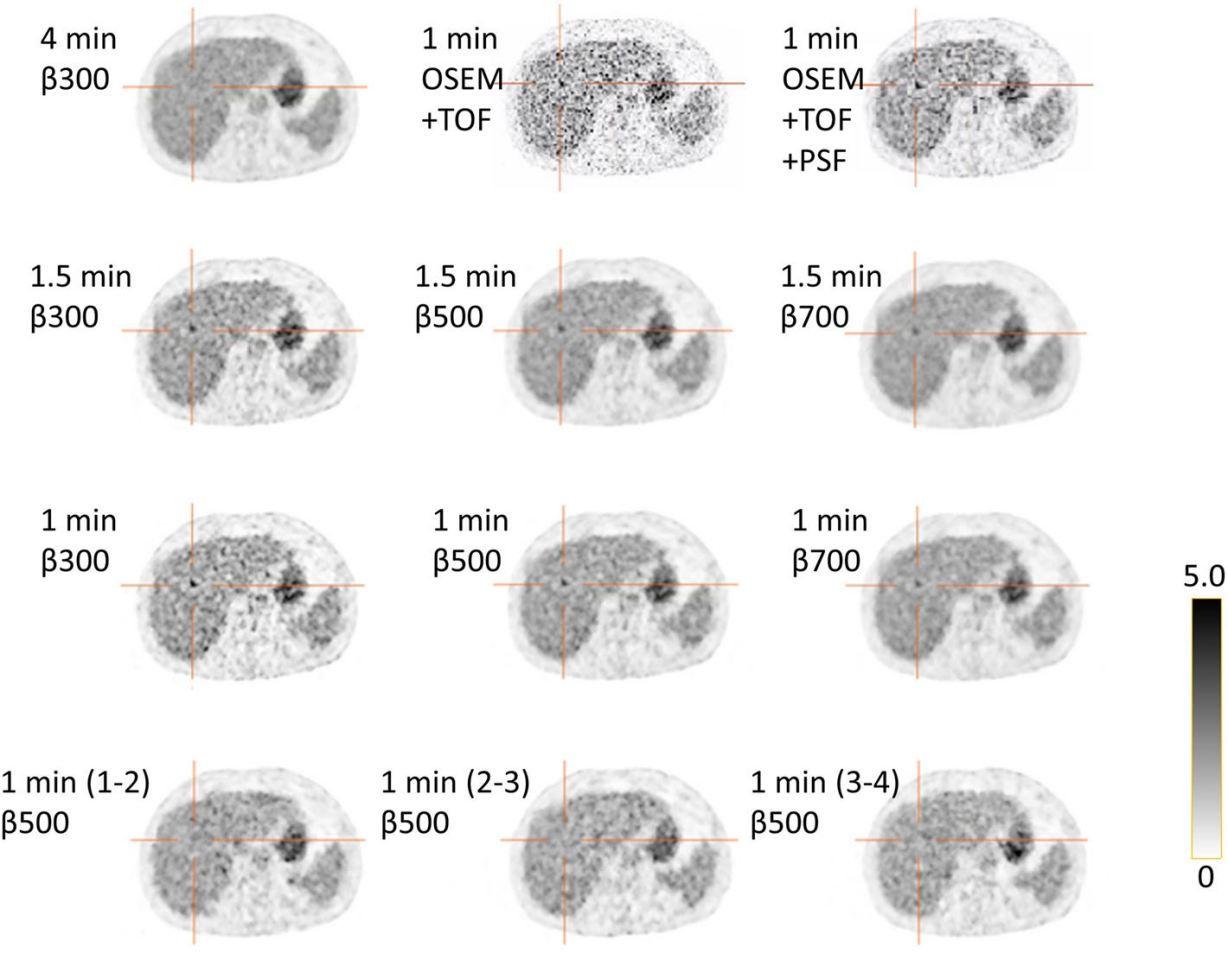
Example of a lesion in the liver not visible in the 4-min reference (upper left corner), to different degrees, visible in the other combinations of time/bed position and *β* values evaluated. The last row shows 1-min reconstructions from the 4-min acquisition for the last 3 min, where the lesion is not visible. OSEM+TOF with and without PSF are shown in the upper row

When the image quality was assessed, a couple of FDG hotspots mimicking lesions were encountered in images obtained with ATs of 4 and 6 but not on the reference AT 16 image. Figures 5 and 6 show examples of such false positive lesions, particularly visible with lower *β* values and an AT of 4. The lesions were only visible in the first-minute series and were also visible on the OSEM+TOF and OSEM+TOF+PSF images.

## Discussion

In this study, we found that the noise level decreased with increasing *β* values and ATs. There was a considerably large difference in SUV_max_ for different *β* values and ATs, whereas SUV_peak_ was more stable. Lesion detectability and image quality were assessed only for ATs of 4 and 6 with *β* of 300–700. Lesion detectability was best for an AT of 6 with a *β* of 500, whereas the largest proportion with acceptable image quality was obtained for an AT of 6 with a *β* of 600.

The number of lesions considered to be uncertain or not detectable for an AT of 4 was higher than that for an AT of 6, especially for lower *β* values. This can be explained by the high noise level in those images. In general, lesions were assessed as uncertain or not detectable when the noise in the surrounding tissue was at the same level as in the lesion or if the lesion was blurred leading to a tracer uptake not exceeding that of the surrounding tissue. In our study, the only combinations of AT and *β* with a sufficient proportion of images of acceptable quality was for an AT of 6 with *β* of 500–600. We have also presented two examples (Figs. 5 and 6) of hotspots seen in images with an AT of 4 and, to a lesser extent, the AT of 6 images but not in the corresponding AT of 16 images. In order to determine if the hotspots were false positives (noise aggregation), we divided the 4 min/bed image into four consecutive 1-min acquisitions. The hotspots were visible in the first 1-min reconstruction but not in the succeeding acquisitions. One could speculate that the patient moved during the later part of the 4-min acquisition and this thereby smoothed the images so that the true lesion was blurred but, as seen in Figs. 5 and 6, this explanation is less likely. By reconstructing the images with OSEM with TOF with and without PSF, we could also establish that it was not the BSREM algorithm that created the false positive uptakes. We, therefore, do not recommend an AT lower than 6. de Groot et al. [17], referenced in the European Association of Nuclear Medicine procedure guidelines for FDG tumor imaging [18], recommend an AT of 7 for PET-CT systems with analog PM tubes. The system studied by de Groot has an overlap of bed positions of 50%, and they suggest that, if the overlap is less than 30%, the AT needs to be increased to 14. However, even though the system in this study only has an overlap of 24%, the results show that the image quality and detectability of lesions will be adequate using ATs ≥ 6.

The *β* value needs to be adjusted depending on the acquisition time. The use of low *β* values requires a high AT (longer acquisition times or higher administered activity), for example, the reference in this study of 4 min/bed position with a *β* of 300. A similar noise level as our reference 4 min/bed position (AT = 16) with *β* of 300 was found for 3 min/bed position (AT = 12) with a *β* of 400, for 2 min/bed position (AT = 8) with a *β* of 500, and for 1.5 min/bed position (AT = 6) with a *β* of 700. For acquisition times of 1 min/bed position (AT = 4), the *β* value needs to be even higher but higher *β* values were not included in this study.

Lesion SUV_max_ varied considerably with both AT and *β* value. This should be considered especially if a low AT and low *β* value are used. Uncertainty in SUV_max_ due to noise could possibly affect therapy response assessments, for example, Deauville scores in patients with lymphoma [19]. SUV_peak_ was more stable (less noise dependent) but requires lesions equal to or larger than 1 cm^3^ to be relevant. In our study, the lesions were approximately 1 cm and thus, at the lower limit of a relevant SUV_peak_. These results demonstrate the importance of using the same acquisition and reconstruction parameters for follow-up examinations.

Lindström et al. [14] evaluated BSREM in a smaller patient population using the same SiPM-based PET-CT as in our study. With an AT of 11.1, they found a best subjective image quality for a *β* of 400 when summarizing seven different aspects of image quality. This is in the same range as the equation in this study yields (AT = 11.1 yields a *β* of 416). Teoh et al. [4] investigated image quality and lesion detectability in oncologic patients examined with ^18F^FDG on a PM-based time-of-flight PET-CT scanner. For an AT of 16, they found an optimal *β* value of 400, which is slightly higher than found in our study, which would yield a *β* of 288. Differences in PET-CT systems and accumulation times, which was 90 min in their study, may affect the results.

A previous study by Howard et al. [16] investigated BSREM versus OSEM in small pulmonary nodules. They found that BSREM increased lesion visual conspicuity and SUV_max_ when using a *β* value of 150 compared with OSEM. With such low *β* values, the noise level is suspected to be very high, if not a high activity or a long acquisition time is used. A high noise level may be acceptable when only small pulmonary nodules are investigated, but not for routine oncological PET-CT examinations. Reynés-Llompart et al. [15] found an optimal *β* of 350 for torso images for an AT of 5. They used a bismuth germanate PET-CT scanner, which is a different detector system than in the present study and without time of flight, which may affect the results.

### Limitations

First, due to the large number of images, only the clinically relevant times per bed position and *β* values were assessed by the nuclear medicine physicians. Ideally, all images would have been evaluated and a scoring system with more steps than simply acceptable/unacceptable image quality would have been used. However, due to limited time, this was not possible. Second, the reconstruction series of 4 min/bed position with a *β* of 300 were considered as the gold standard but this does not necessarily represent the truth. Third, there was a mix of non-contrast-enhanced and contrast-enhanced CTs used for attenuation correction. Previous studies [20, 21] have found higher SUV in PET images when contrast-enhanced CTs are used for attenuation correction, although with only small differences.

## Conclusion

For oncologic whole-body ^18^F-FDG examinations on a SiPM-based PET-CT, we propose using an AT of 6 with a *β* value of 500–600 in order to achieve high image quality and lesion detection rate. We do not recommend short ATs (< 6), as the risk of false positive uptakes due to noise increases.

## Data Availability

The datasets used and/or analyzed during the current study are available from the corresponding author on reasonable request.

## Abbreviations

AT: Activity time product
BSREM: Block-sequential regularized expectation maximization
FDG: Fluorodeoxyglucose
IQR: Interquartile range
OSEM: Ordered subset expectation maximization
PET-CT: Positron emission tomography with computed tomography
PM: Photomultiplier
PSF: Point spread function
ROI: Region of interest
SD: Standard deviation
Si: Silicon
SUV: Standardized uptake value
TOF: Time of flight

## References

[1] Roncali E, Cherry SR (2011). Application of silicon photomultipliers to positron emission tomography. Ann Biomed Eng.

[2] Hsu David F.C., Ilan Ezgi, Peterson William T., Uribe Jorge, Lubberink Mark, Levin Craig S. (2017). Studies of a Next-Generation Silicon-Photomultiplier–Based Time-of-Flight PET/CT System. Journal of Nuclear Medicine.

[3] Wagatsuma K, Miwa K, Sakata M, Oda K, Ono H, Kameyama M (2017). Comparison between new-generation SiPM-based and conventional PMT-based TOF-PET/CT. Phys Med.

[4] Teoh EJ, McGowan DR, Macpherson RE, Bradley KM, Gleeson FV (2015). Phantom and clinical evaluation of the Bayesian penalized likelihood reconstruction algorithm Q. Clear on an LYSO PET/CT system. J Nucl Med.

[5] Ross SQ (2014). Clear, GE Healthcare. White paper.

[6] Ahn S, Fessler JA (2003). Globally convergent image reconstruction for emission tomography using relaxed ordered subsets algorithms. IEEE Trans Med Imaging.

[7] de Pierro AR, Beleza Yamagishi ME (2001). Fast EM-like methods for maximum “a posteriori” estimates in emission tomography. IEEE Trans Med Imaging.

[8] Nuyts J, Fessler JA (2003). A penalized-likelihood image reconstruction method for emission tomography, compared to postsmoothed maximum-likelihood with matched spatial resolution. IEEE Trans Med Imaging.

[9] Ahn S, Ross SG, Asma E, Miao J, Jin X, Cheng L (2015). Quantitative comparison of OSEM and penalized likelihood image reconstruction using relative difference penalties for clinical PET. Phys Med Biol.

[10] Wangerin KA, Ahn S, Wollenweber S, Ross SG, Kinahan PE, Manjeshwar RM (2017). Evaluation of lesion detectability in positron emission tomography when using a convergent penalized likelihood image reconstruction method. J Med Imaging (Bellingham).

[11] Parvizi N, Franklin JM, McGowan DR, Teoh EJ, Bradley KM, Gleeson FV (2015). Does a novel penalized likelihood reconstruction of 18F-FDG PET-CT improve signal-to-background in colorectal liver metastases?. Eur J Radiol.

[12] Teoh EJ, McGowan DR, Schuster DM, Tsakok MT, Gleeson FV, Bradley KM (2018). Bayesian penalised likelihood reconstruction (Q.Clear) of (18) F-fluciclovine PET for imaging of recurrent prostate cancer: semi-quantitative and clinical evaluation. Br J Radiol.

[13] Ter Voert E, Muehlematter UJ, Delso G, Pizzuto DA, Muller J, Nagel HW (2018). Quantitative performance and optimal regularization parameter in block sequential regularized expectation maximization reconstructions in clinical (68) Ga-PSMA PET/MR. EJNMMI Res.

[14] Lindstrom E, Sundin A, Trampal C, Lindsjo L, Ilan E, Danfors T (2018). Evaluation of penalized-likelihood estimation reconstruction on a digital time-of-flight PET/CT scanner for (18) F-FDG whole-body examinations. J Nucl Med.

[15] Reynes-Llompart G, Gamez-Cenzano C, Vercher-Conejero JL, Sabate-Llobera A, Calvo-Malvar N, Marti-Climent JM (2018). Phantom, clinical, and texture indices evaluation and optimization of a penalized-likelihood image reconstruction method (Q.Clear) on a BGO PET/CT scanner. Med Phys.

[16] Howard BA, Morgan R, Thorpe MP, Turkington TG, Oldan J, James OG (2017). Comparison of Bayesian penalized likelihood reconstruction versus OS-EM for characterization of small pulmonary nodules in oncologic PET/CT. Ann Nucl Med.

[17] de Groot EH, Post N, Boellaard R, Wagenaar NR, Willemsen AT, van Dalen JA (2013). Optimized dose regimen for whole-body FDG-PET imaging. EJNMMI Res.

[18] Boellaard R, Delgado-Bolton R, Oyen WJ, Giammarile F, Tatsch K, Eschner W (2015). FDG PET/CT: EANM procedure guidelines for tumour imaging: version 2.0. Eur J Nucl Med Mol Imaging.

[19] Barrington SF, Kluge R (2017). FDG PET for therapy monitoring in Hodgkin and non-Hodgkin lymphomas. Eur J Nucl Med Mol Imaging.

[20] Berthelsen AK, Holm S, Loft A, Klausen TL, Andersen F, Hojgaard L (2005). PET/CT with intravenous contrast can be used for PET attenuation correction in cancer patients. Eur J Nucl Med Mol Imaging.

[21] Aschoff P, Plathow C, Beyer T, Lichy MP, Erb G, Oksuz MO (2012). Multiphase contrast-enhanced CT with highly concentrated contrast agent can be used for PET attenuation correction in integrated PET/CT imaging. Eur J Nucl Med Mol Imaging.

